# Initial systemic inflammatory state perturbs exercise training adaptations in elite Taekwondo athletes

**DOI:** 10.1371/journal.pone.0176140

**Published:** 2017-04-19

**Authors:** Chung-Yu Chen, Yi-Hung Liao, Chun-Chung Chou, Yu-Chi Sung, Shiow-Chwen Tsai

**Affiliations:** 1 Department of Exercise and Health Sciences, University of Taipei, Taipei City, Taiwan; 2 Department of Exercise and Health Science, National Taipei University of Nursing and Health Sciences, Taipei City, Taiwan; 3 Physical Education Office, National Taipei University of Technology, Taipei City, Taiwan; 4 Department of Chinese Martial Arts, Chinese Culture University, Taipei City, Taiwan; 5 Department of Sports Sciences, University of Taipei, Taipei City, Taiwan; Universitat de les Illes Balears, SPAIN

## Abstract

**Purpose:**

This study examined ten-week TKD-specific training effects on aerobic capacity, body composition, hormone responses and hematological parameters in elite TKD athletes with varied initial inflammatory states.

**Methods:**

Twenty-two elite college TKD athletes were divided into two groups according to their initial neutrophils-to-lymphocytes ratio (NLR) values: Low NLR (N = 11, 9M/2F, age: 21.6 ± 1.0 yrs; NLR: 1.3 ± 0.2) and High NLR (N = 11, 8M/3F, age: 22.0 ± 0.7 yrs, NLR: 2.5 ± 1.3), and participated in a 10-week TKD-specific training program. Aerobic capacity, body composition, hormonal responses and hematological parameters were measured at baseline and 10-weeks after TKD training.

**Results:**

VO_2max_ and shuttle run distance were significantly increased in both groups after training. However, the degree of improvement was greater in the Low NLR group than in the High NLR group. After 10-weeks of exercise training, the High NLR group presented markedly higher fat mass percentage and visceral fat area and significantly lowers DHEA-S to cortisol ratio (D/C ratio) than the Low NRL group. The post-training NLR was negatively correlated with the D/C ratio. Neutrophil counts and NLR were still significantly higher in the High NLR group after training.

**Conclusions:**

This study provides new evidence that young elite TKD athletes with slightly high baseline systemic inflammatory state appear to perturb adaptations to exercise training.

## Introduction

Taekwondo (TKD) is a traditional martial art originated from Korea. This combat sport was initially aimed to train military soldiers in combat skills for self-defense. According to the World Taekwondo Federation (WTF) and International Taekwondo Federation (ITF) reports, there are over 80 million people in 180 countries that practice this sport worldwide. This combat sport became an official Olympic sport in the 2000 Sydney Olympic Games. The TKD training program contains systematic, chronic, progressive activities [1]. TKD requires high aerobic capacity and anaerobic power, rapid movement skills, high muscle strength, excellent body composition and overall agility [2, 3]. Intensified physical and specific training strategies are important for coaches and scientists to enhance the technical skills and competitive performance in TKD athletes.

For highly competitive sports, obtaining the best exercise training outcomes is a primary concern for both coaches and athletes. Experienced and elite TKD athletes have been reported to have better aerobic performance and muscle strength training effects compared with novice or non-practitioners [4]. It was also reported that the mean VO_2max_ value in experienced male TKD athletes was lower than that of novice TKD practitioners [5, 6]. This discrepancy in results implies that the internal physiologic mediator plays an important role in the exercise training outcome. Recent investigations have shown that inflammation plays a critical role in the adaptation to consecutive physiological stress [7]. For example, temporally inducing cytokines, monocyte, and macrophage-released by local inflammation is considered a trigger that regulates the muscle repair and regeneration process in response to exercise [7, 8].

The localization and systemic inflammation seem to play critical roles in the cardiopulmonary capacity [9] and the process of exercise-induced adaptations [10, 11, 12, 13, 14]. Previous evidence reveals that local infusion non-steroidal anti-inflammatory drug (NSAIDs) inhibits satellite cell proliferation in young human skeletal muscle after eccentric exercise [10]. Meanwhile, Mackey et al. demonstrated the negative regulatory effect of NSAIDs on exercise-induced human myogenic precursor cell responses [11]. These results suggest that NSAIDs administration during exercise perturbed local muscle inflammation and attenuated the muscle adaptation. It’s notable that aging leads to the immunological change and is accompanied by increasing chronic inflammatory state [12]. Recently studies have demonstrated that long-term NSAIDs administration significantly improves skeletal muscle adaptations induced by resistance training in elderly adults [13]. Likewise, Rieu et al. reported that chronic ibuprofen administration markedly increases muscle mass by reducing the low-grade systemic inflammation in old rats [14]. These findings imply a possibility that reduced the systemic inflammation by NSAIDs may sensitize the exercise-induced local skeletal muscle inflammation and the subsequent muscle rebuild and adaptation. Therefore, we hypothesize the initial systemic inflammatory status may play a critical role in the adaptations in response to long-term exercise training.

Among the currently used biomarkers for evaluating systemic inflammation (e.g. C-reactive protein, tumor necrosis factor-α, etc.) are more expensive and inconvenient. The ratio of neutrophils-to-lymphocytes (NLR), calculated according to the results of regular hematological examination, has been recently recognized as a practical and easy biomarker to assess the systemic inflammatory status in human body. Furthermore, the NLR is highly correlated to the circulating level of C-reactive protein (CRP) [15]. NLR is a robust biomarker of inflammatory, infectious, and postoperative complications in the current clinical usage [16, 17, 18, 19, 20]. Also, Sultan et al. investigating the role of inflammation in cardiopulmonary capacity in preoperative patients, reported that the individuals with higher NLR exhibited relatively lower anaerobic threshold values (AT) and that their preoperative NLR was strongly associated with their AT values [9]. In this regard, our previous study selected the NLR to reflect the systemic inflammatory status in combative sport athletes [21]. As stated above, because the systemic inflammation may perturb the exercise-induced adaptive responses, we then used this convenient biomarker as a pre-screen tool to investigate whether the exercise training adaptation in elite TKD athletes would be affected by the varied initial degrees of systemic inflammatory states. Taken together, the purpose of this study was to determine the effects of a ten-week TKD-specific training on aerobic capacity, body composition, hormone responses and hematological parameters in elite TKD athletes with varied initial degrees of systemic inflammatory states.

## Methods

### Participants

Twenty-two elite college Taekwondo athletes (National Division I category) were voluntarily participated in this study. After the initial pre-test, their blood hematological parameters were examined, and the neutrophil/lymphocyte ratio (NLR) values were obtained. The NLR value of these young athletes were averaged at 1.9 ± 1.1, which was under the normal value (0.78~ 3.53) [22]. The individual NLR values were blinded to the participants to minimize the possible confounding factors to the exercise training, and then all participants were divided into two groups based on their baseline NLR: Low NLR group (n = 11, 9 male and 2 female, age: 21.6 ± 1.0 years, NLR: 1.3 ± 0.2) and High NLR group (n = 11, 8 male and 3 female, age: 22.0 ± 0.7 years, NLR: 2.5 ± 1.3). All participants were black belt holders, having at least 8 years competitive experience in this sport. All participants completed a health screening questionnaire and had no history of musculoskeletal injuries or other metabolic disorders. Participants were provided a written informed consent after the experimental purpose, procedures and possible potential risks were explained. This study was approved by the Institute Review Board (IRB) of the University of Taipei (IRB, Taipei City, Taiwan).

### Experimental design

After an overnight fast (10 h), a venous blood sample was collected and aerobic capacity, body composition were measured for both pre and post ten-week exercise training. Participants were restricted from consuming any form of anti-inflammatory drug or antioxidants to minimize the individual variability in detecting inflammatory state during the training period. Also, participants were requested to maintain the same dietary pattern during intervention. Blood samples were used to determine hormonal responses (DHEA-S and cortisol) and hematological parameters (neutrophils and lymphocytes).

### TKD-specific training program

The ten-week exercise training program consisted of dynamic warm up (jogging and stretching; training duration: 20 min, frequency: 5 times/week), fundamental motor skills practice (basic gait technique, weight transfer technique; training duration: 20 min, frequency: 5 times/week), specific technical practice (punches, blocks, kicks, twists; training duration: 30 min, frequency: 5 times/week), aerobic training (training duration: 30 min, frequency: 3 times/week), specific strength training (front leg lift and side leg lift; training duration: 20 min, frequency: 2 times/week), and specific flexibility training (training duration: 20 min, frequency: 2 times/week). The overall exercise period duration per each individual was approximately 2 hours. All training procedures were supervised by personal trainers and coaches to ensure training quality and consistency.

### Body compositions

The height and body weight of all participants were measured after an overnight fast. We also measured the body composition (visceral fat area, muscle mass and percentage of fat mass) using a bio-impendence body composition analyzer (InBody 720, InBody Co., Ltd, Seoul, Korea).

### Aerobic capacity measurement

The maximal aerobic capacity in this study was determined using a 20-meter shuttle run test [23]. During the test, participants ran a 20-meter distance, and turned and ran back. Participants followed the sound signals from a prerecorded tape. Each emitted sound guided the participant to touch the parallel lines spaced 20 meters apart. The initial speed began at 8.5 km/h and gradually increased 0.5 km/h at each minute. When a participant could no longer follow the signal pace in two consecutive times, the shuttle was recorded and the test ended. According to the test, the predicted maximal oxygen consumption (VO_2max_; ml/kg/min) was calculated using the following formula: 31.025 + 3.238 × speed (km/h)– 3.248 × Age (yrs) + 0.1536 × speed (km/h) × Age (yrs).

### Hematology and hormones analysis

Fasting venous whole blood samples (5 ml) were collected in tubes with EDTA to analyze hematological profiles (i.e. neutrophils and lymphocytes) using an automated hematology analyzer (Sysmex XT-2000, Sysmex Corp., Kobe, Japan) in the light of manufacturer’s instruction. The remaining whole blood samples were centrifuged at 4°C, 3000 rpm for 10 min to obtain plasma samples. The plasma samples were then stored at -80°C until analyzed. Cortisol and dehydroepiandrosterone-sulfate (DHEA-S) were evaluated using commercially available enzyme-linked immunosorbent assay (ELISA) kits (Cayman Chemical Co., Ann Arbor, MI, USA and IBL Inc., Hamburg, Germany) according to the manufacturer’s instructions. All ELISA assays were read using a TECAN Genios ELISA reader (Salzburg, Austria). The optical density was used to determine the amount of each hormone in these plasma samples.

### Statistical analysis

All data were presented as mean ± standard deviation (Mean ± SD). For statistical analysis and graph, SPSS 18.0 software (IBM SPSS statistics for Windows, New York, USA) and GraphPad Prism 5.0 (GraphPad software Inc., La Jolla, CA, USA) were used. The Shapiro-Wilk test was used to analyze the normal distribution for all variables. To compare the training effect of group assignment on each outcome, we use a mixed design analysis of variance with low/high-NLR value as the between-subject factor and pre/post-training as the within-subject factor. In this study, the values of NLR and D/C ratio were log transformed for analysis in SPSS due to the skewed data distribution in these two variables. Pearson’s correlation coefficient test was applied to assess the correlation between D/C ratio and NLR. The alpha level was set at 0.05 (*p* < .05) for accepting statistical difference for all comparisons.

## Results

The baseline physical and hematologic characteristics between Low and High NLR groups are shown in Table 1. There were no differences in age, height, body weight, percentage of fat mass, muscle mass, visceral fat area, VO_2max_, shuttle run distance, white blood cell (WBC), DHEA-S, cortisol, and DHEA-S to cortisol (D/C ratio) between both experimental groups. The neutrophil counts and ratio of neutrophil to lymphocyte (NLR) were significantly higher in High NLR group at baseline. Significantly lower lymphocyte counts were observed in the High NLR group than in the Low NLR group.

**Table 1:** Baseline physical and hematologic characteristics of elite Taekwondo athletes.

Measurements	Low NLR group	High NLR group	Sig
Number (M;F)	11 (9M; 2F)	11 (8M; 3F)	
Age (year)	21.6 ± 1.0	22.0 ± 0.7	*P* = .18
Height (cm)	173.9 ± 6.2	174.5 ± 7.2	*P* = .37
Body weight (kg)	66.9 ± 8.8	72.3 ± 13.5	*P* = .14
Fat mass (%)	16.4 ± 4.9	19.2 ± 6.7	*P* = .15
Muscle mass (kg)	31.5 ± 4.2	31.8 ± 5.8	*P* = .45
Visceral fat area (cm^2^)	42.2 ± 16.1	50.7 ± 17.3	*P* = .12
VO_2_ max (ml/kg/min)	46.2 ± 4.7	42.3 ± 7.4	*P* = .08
Shuttle run distance (m)	1578.0 ± 225.6	1462.0 ± 389.1	*P* = .20
WBC (number/ml)	6032.7 ± 964.2	6659.1 ± 1593.9	*P* = .14
Neutrophil (%)	49.8 ± 5.2	63.0 ± 6.4	*P*< .001
Lymphocyte (%)	39.8 ± 5.1	28.0 ± 6.2	*P*< .001
NLR	1.3 ± 0.2	2.5 ± 1.3	*P* = .004
DHEA-S (ug/dL)	363.3 ± 99.7	302.3 ± 131.5	*P* = .12
Cortisol (ug/dL)	23.9 ± 7.2	23.8 ± 8.5	*P* = .48
D/C ratio	15.9 ± 4.3	14.9 ± 10.3	*P* = .39

Values are expressed as mean ± SD. WBC: White blood cell, NLR: Neutrophil to lymphocyte ratio, D/C ratio: DHEA-S to cortisol ratio.

**Fig 1:**
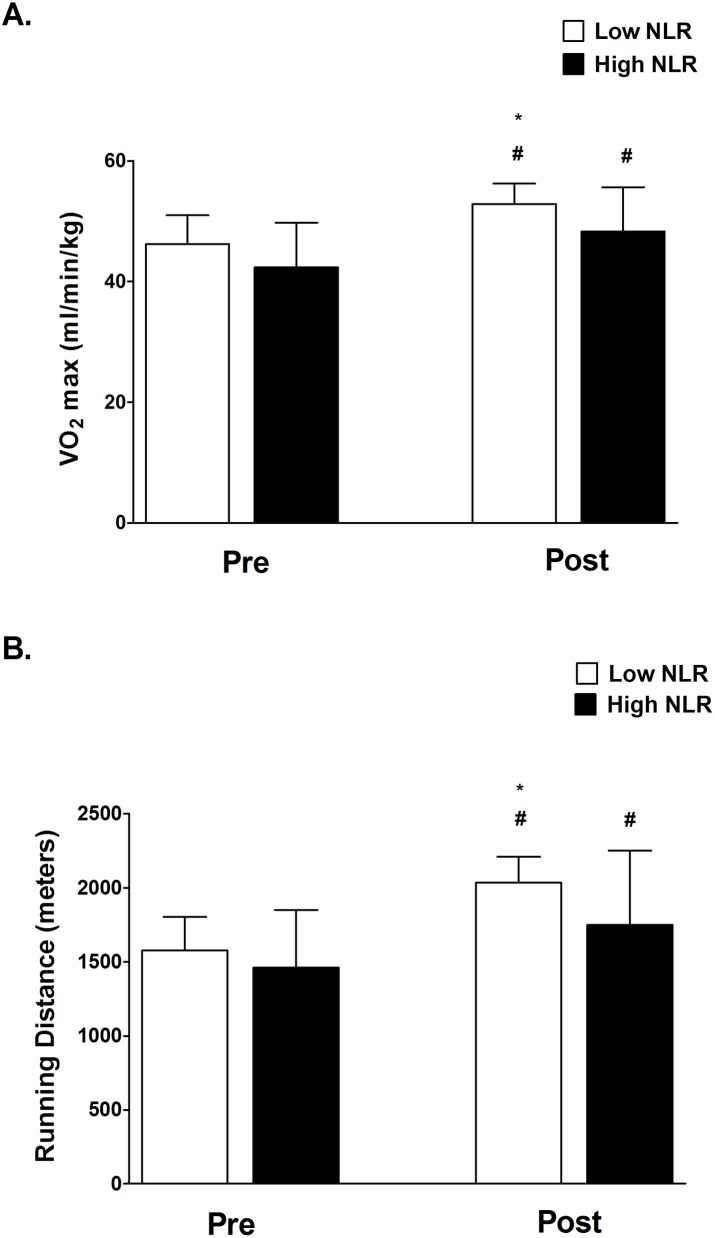
**Changes in VO**_**2**_**max (A) and shuttle run distance (B) between Low and High NLR groups at Pre and Post-exercise training.** Values are expressed as mean ± SD. *Significant difference between Low and High NLR groups (*p* < .05). # Significant difference between Pre and Post (*p* < .05).

**Fig 2:**
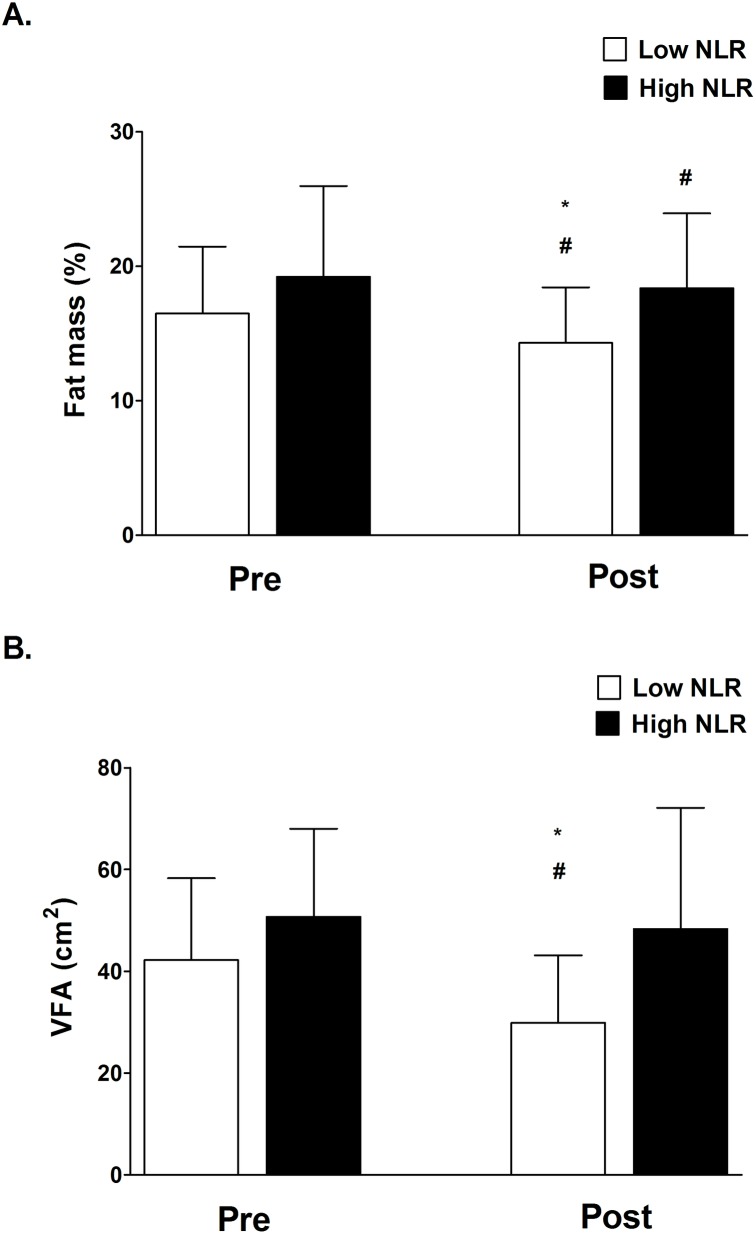
Changes in body composition between Low and High NLR groups at Pre and Post-exercise training. High NLR group show higher fat mass percentage (A) and visceral fat area (B) after training. Values are expressed as mean ± SD. *Significant difference between Low and High NLR groups (*p* < .05). # Significant difference between Pre and Post (*p* < .05).

**Fig 3:**
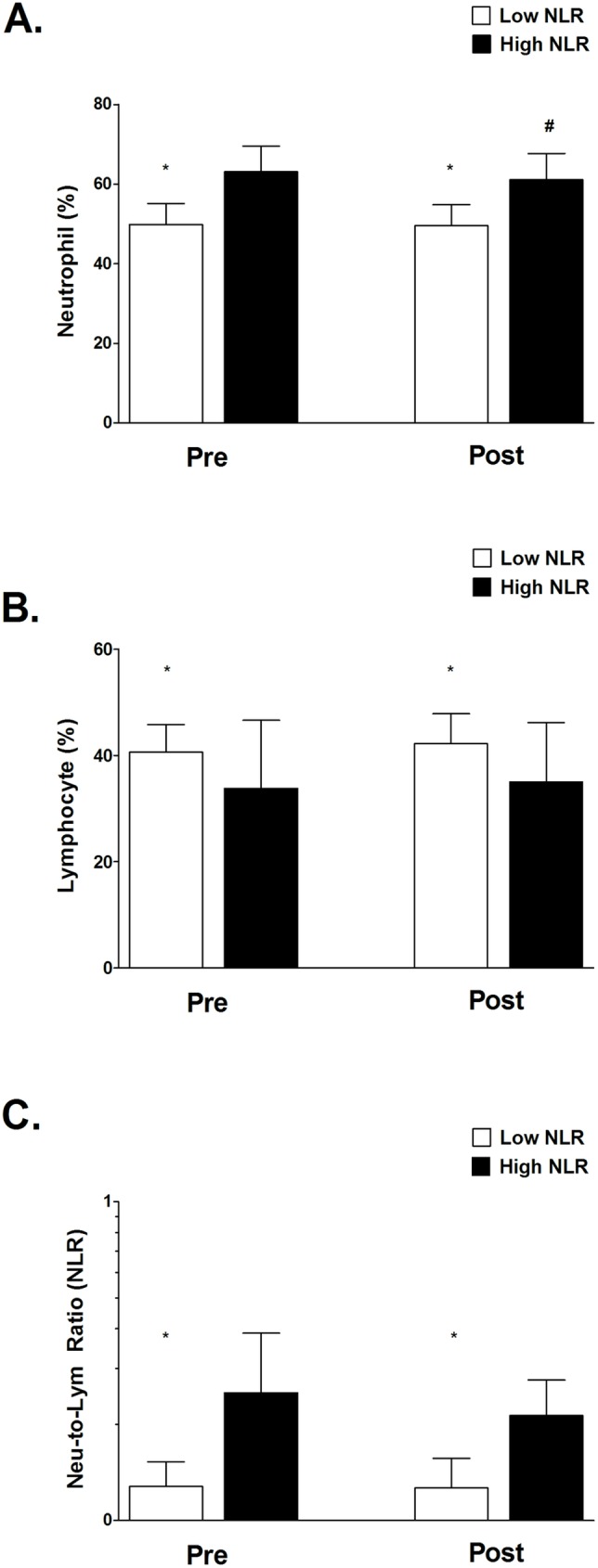
Changes in hematological profiles and inflammatory index between Low and High NLR groups at Pre and Post-exercise training. Compared to Low NLR group, High NLR group shows higher neutrophil counts (A) and lower lymphocyte counts (B) at pre and post training. High NLR level group shows higher neutrophil to lymphocyte ratio (NLR) at pre and post training (C). Values are expressed as mean ± SD. *Significant difference between Low and High NLR groups (*p* < .05). # Significant difference between Pre and Post (*p* < .05).

**Fig 4:**
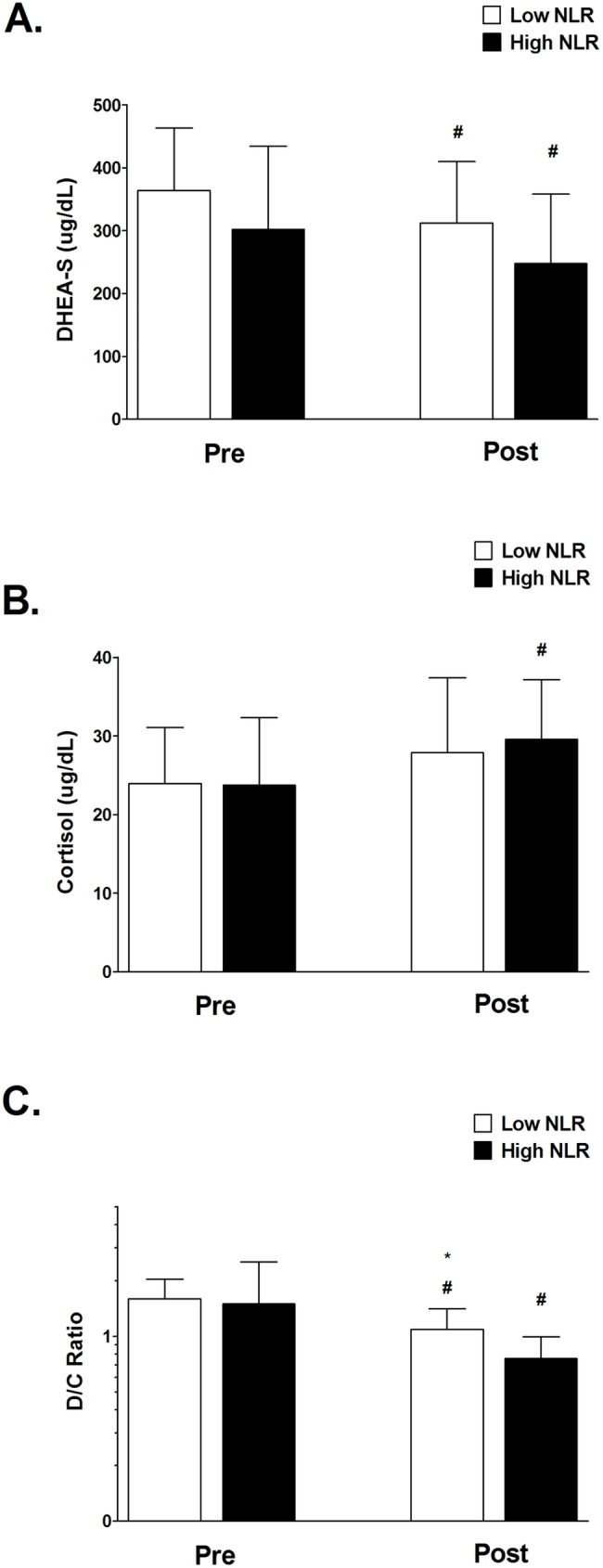
Changes in DHEA-S and cortisol levels between Low and High NLR groups at Pre and Post-exercise training. No difference in DHEA-S (A) and cortisol (B) levels in both groups after training. Compared to Low NLR group, High NLR group shows lower D/C ratio after ten-week training (C). Values are expressed as mean ± SD. *Significant difference between Low and High NLR groups (*p* < .05). # Significant difference between Pre and Post (*p* < .05).

**Fig 5:**
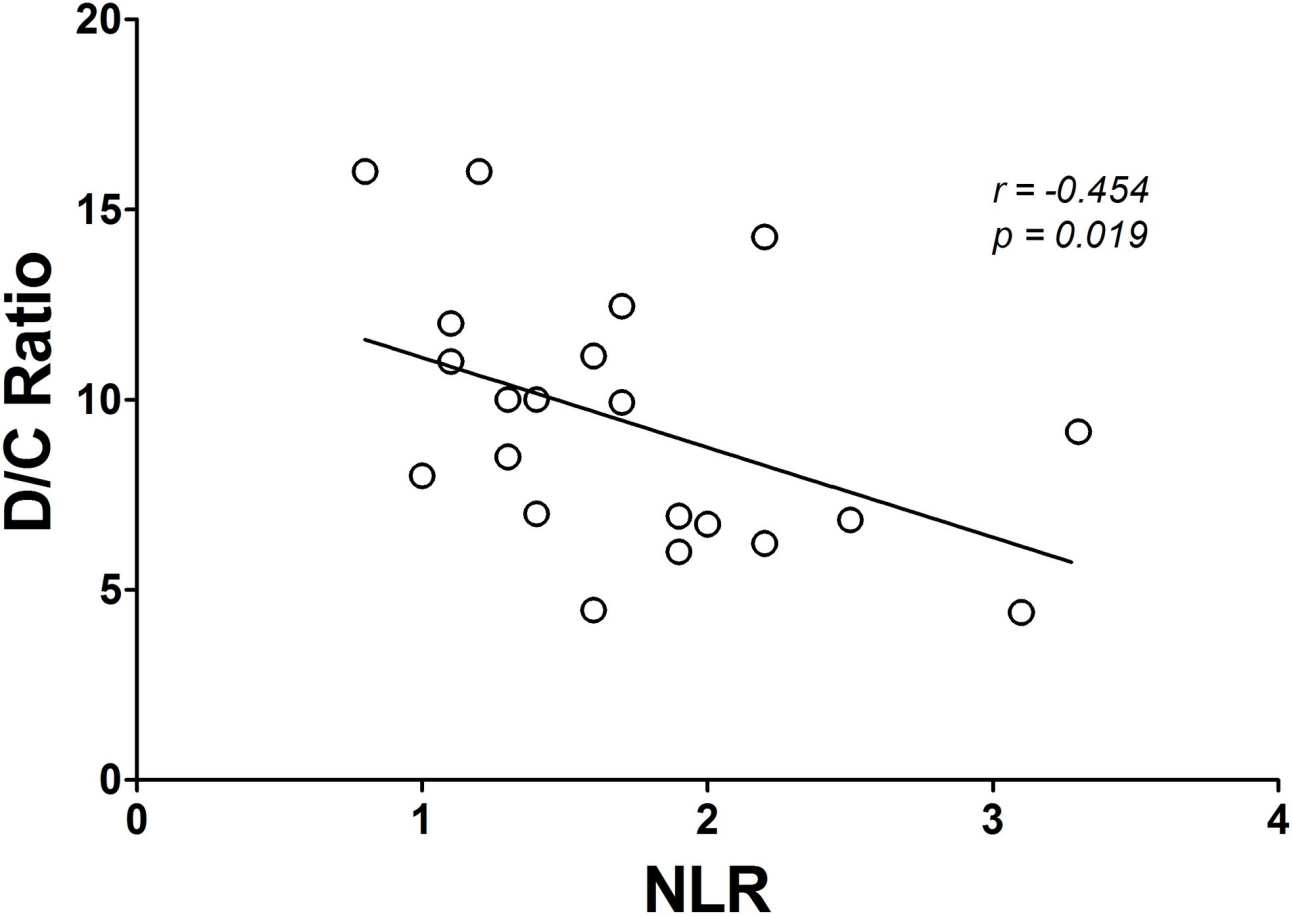
The correlation between post-training NLR level and D/C ratio in TKD athletes. NLR level was negatively correlated with the D/C ratio (*r* = -0.454, *p* = 0.019). This result indicates that the anabolic capacity is associated with the NLR level.

After ten weeks of TKD-specific training, the VO_2max_ value and shuttle run distance were significantly enhanced in both Low and High NLR groups. However, we also observed the degree of improvement in VO_2max_ and shuttle run distance in the Low NLR group was greater than in the High NLR group (Fig 1A and Fig 1B). Fig 2 shows the changes in body composition for both Low and High NLR groups at pre and post exercise training. Exercise training significantly reduced the percentage of fat mass in both Low and High NLR groups (Fig 2A), however, the reduction of visceral fat area (VFA) was only observed in Low NLR group. Furthermore, compared to Low NLR group, High NLR group showed significantly higher percentage of fat mass and VFA after training. The muscle mass value was not different between the Low and High NLR groups. Fig 3 shows the changes in hematological profiles and inflammatory index for both Low and High NLR groups at pre and post exercise training. The neutrophil counts in the High NLR group were significantly higher than in the Low NLR group at pre and post exercise training (Fig 3A). Furthermore, the lymphocyte counts in the High NLR group were significantly lower than the Low NLR group at pre and post training (Fig 3B). Fig 3C shows the change of NLR. The NLR was still significantly higher in the High NLR group than the Low NLR group after training. The levels of DHEA-S and cortisol at pre and post exercise training are shown in Fig 4A and 4B. Ten-week exercise training significantly reduced the DHEA-S level in both Low and High NLR groups. The cortisol level was found significantly increased in High NLR group after training. Additionally, ten-week exercise training significantly reduced the D/C ratio in both Low and High NLR groups. The High NLR group displayed a markedly lower D/C ratio compared to those Low NLR group (Fig 4C). The correlation between NLR level and D/C ratio after training is shown in Fig 5. The NLR level was negatively correlated with the D/C ratio (*r* = -0.454, *p* = 0.019).

## Discussion

Long-term NSAID supplementation has been shown to produce better exercise training effect on increasing muscle mass and strength by reducing systemic inflammation in older individuals [13, 14]. However, it is not completely understood whether initial systemic inflammatory state would affect the exercise-training adaptation in young elite TKD athletes. The major findings of this study are that ten-week TKD-specific training enhances aerobic capacity (VO_2max_ and shuttle running distance) in both Low and High NLR groups, but the degree of improvement in aerobic capacity in the Low NLR group is superior to that in the High NLR group. In addition, the High NLR group reveals a higher percentage of fat mass and visceral fat value after exercise training compared to the Low NLR group. We also observed that the D/C ratio, an index of anabolic capacity, was significantly lower in the High NLR group compared to Low NLR group after ten-week exercise training. Exercise training slightly reduced the NLR in the High NLR group, whereas the NLR level was still significantly higher in the High NLR group than the Low NLR group. Our study was the first study to demonstrate the slight higher initial systemic inflammation would attenuate the effectiveness of exercise training adaptation in these young elite TKD athletes.

Inflammation is a physiological response to recruit bioresources to the damage location to facilitate the repair process. This response has been recently considered a fundamental process to induce training adaptation in response to exercise [7, 8]. Although previous studies have shown that NSAIDs treatment negatively affect the muscle satellite cell numbers and activity by reducing prostaglandin synthesis after exercise [10, 11]. However, study from Trappe and colleagues observed that 12 weeks knee extensor progressive resistance exercise training with NSAIDs supplementation significantly increased muscle mass and strength than placebo in older adults [13]. These discrepant results appear to imply that reduced systemic inflammation by NSAIDs administration during exercise training could bring better benefits to exercise training adaptation. This phenomenon might account for the varied levels of training adaptations following exercise training, whereas this theory has not been wildly supported by the existing evidence. Moreover, it has not been clear whether pre-training inflammatory levels would affect the consequence of intensified TKD-specific training.

NLR was recently considered as inflammatory, infectious, and postoperative complications biomarkers [9, 16,17, 18, 19, 20] and was found to be highly correlated to the circulating level of C-reactive protein [15], suggesting the NLR was a sensitive and reliable biomarker to evaluate the systemic inflammatory state during exercise training. More recently, NLR is also used to access the systemic inflammatory state in athletes, diabetic and colorectal cancer patients [16, 21, 24]. We therefore selected this inflammatory biomarker to determine the systemic inflammatory level of participants in this study. Previous study had reported that the higher NLR strongly associated with lower cardiopulmonary capacity in preoperative colorectal patients [9]. In line with the study conducted by Sultan et al. (2014), here we also observed that the athletes with initial higher NLR level exhibited relatively less training-enhanced aerobic capacity compared with those with initially lower NLR level. Of note, our recent findings provided new evidence that the initial low-grade inflammatory status did perturb the subsequent training outcomes in these combative sport athletes.

Regular exercise training has been proven to markedly reduce the circulating level of pro-inflammatory cytokines: tumor necrosis factor-α (TNF-α), CRP, and interleukin 6 (IL-6) [25, 26, 27], suggesting an anti-inflammatory effect from long-term regular training. Here, we found that the NLR level in High NLR group was not affected even though underwent 10-week TKD training (Fig 3C). This inconsistent result was possibly related to higher body fat mass and visceral fat was observed in High NLR group after training. Festa et al. reported that body fat accumulation is strongly associated with circulating levels of CRP and fibrinogen [28]. Likewise, our previous study indicated that the increased NLR was related to body fat mass accumulation after 8-week detraining in elite TKD athletes [21], which is in line with our present finding that NLR remains at high levels in the High NLR group following 10-week training. Moreover, previous evidence revealed that 9 months of aerobic exercise training markedly reduced the CRP level in participants preparing for a marathon [26]. Note that the training program in this study only lasted for ten weeks, thus this discrepancy between our and previous study on systemic inflammatory state could also be possibly due to different exercise training duration in that model.

Previous evidence indicates that high aerobic capacity is essential to sustain high-intensity activity and better recovery capacity during competitive events [29, 30, 31]. We observed that ten-week TKD exercise training significantly enhanced VO_2max_ and shuttle running distance in both Low and High NLR groups, which was in line with previous evidence on improving the aerobic capacity after 12-week typical TKD training [32]. However, we observed that the athletes with higher initial systemic inflammatory state displayed relatively less aerobic capacity improvement after TKD-specific training (Fig 1A & 1B). It has been well established that exercise training increases aerobic capacity through elevating muscular mitochondria content, capillary density, and muscle mass [33, 34, 35]. These physiological changes require local muscle inflammation to trigger muscle repair and regeneration [8]. Additionally, chronic NSAID intervention has been reported to elicit better improvement in muscle mass and strength in the elderly [13], suggesting that the well-controlled systemic inflammation could be critical to exercise-induced adaptation. Therefore, we speculate that higher systemic inflammation might perturb the local muscle repair/rebuild process and the subsequent aerobic capacity improvement after training.

Another possibility accounts for the high systemic inflammation reduced training adaptation might be due to the poor anabolic capacity. Previous studies showed that the higher physiological stress reduces anabolic capacity reflected by high catabolic hormone levels (such as cortisol) and low anabolic hormone levels (such as testosterone or DHEA-S) [36, 37, 38], likely reduced training adaptation. The training recovery and tissue regeneration requires these anabolic hormones to facilitate the repair process [39]. These changes are in line with our finding that low D/C ratio in athletes with higher inflammatory state (Fig 4C & Fig 5). Although previous evidences verify our current findings, yet we did not detect the causal relationships between initial systemic inflammatory state and anabolic capacity in this study. It would be an interesting research question whether the inflammation state perturbs the anabolic/catabolic balance and subsequent adaptation in response to exercise training.

### Practical applications

To understand how the potential internal physiologic mediators regulate the adaptation in response to long-term exercise training is quite important for coaches and sports scientists to develop practical strategies to enhance the athlete’s performance. We found that the athletes with relatively higher initial systemic inflammation exhibited less fat mass reduction and aerobic capacity adaptation even after ten-week TKD-specific training. The higher initial inflammatory state perturbation appears to be associated with the reduction in anabolic capacity, which might explain why the fewer adaptive responses after training. These data suggest that if the initial systemic inflammatory state could be substantially lowered and controlled by dietary manipulation or ergogenic supplements before performing regular periodic training, the competitive combat athletes may obtain better training adaptations and sport performance. Our present findings provide possible explanations for the varying degrees of adaptive responses to programmed exercise training. Also, this obtained information would be significant for both coaching science teams and health management professionals to develop practical interventions to eliminate the adverse impacts of slightly higher baseline inflammation on training adaptations in this special population.

## Conclusions

This study demonstrates that elite TKD athletes with slight higher baseline NLR level exhibit relatively less adaptive response on aerobic capacity after 10 weeks TKD-specific training. The percentage of fat mass, visceral fat area, and NLR level were observed higher in the High NLR group than in the Low NLR group after training. We also found that the D/C ratio declined in the High NLR group after training. This suggests that fewer improved exercise-adaptive benefits occurred for the High NLR group might be the result of a higher systemic inflammation state and poor anabolic capacity.
